# Supplementary material for: Systematic Review of the Potential of MicroRNAs in Diffuse Large B Cell Lymphoma
Source: Cancers (Basel). 2019 Jan 26;11(2):144. doi: 10.3390/cancers11020144 (PMC6406874; doi:10.3390/cancers11020144)
Supplement: Supplementary file 1 [file cancers-11-00144-s001.pdf]

cancers

MDPI

Supplementary Materials: Systematic Review of the Potential Of Micrornas in Diffuse Large B Cell Lymphoma

Ane Larrabeiti-Etxebarria, Maria Lopez-Santillan, Borja Santos-Zorrozua, Elixabet Lopez-Lopez and Africa Garcia-Orad

Table S1. Differentially expressed microRNAs in DLBCL patients compared with healthy control individuals.

Significant miRNAs	Result	n DLBCL	n Control	Sample source	Method	Nº miRNAs	Reference
miR-155	up	29	32 (RLH)	Tissue	qRT-PCR	1	Li 2017 [1]
miR-4532	up	5	4 (RLH)	Tissue	nanostring	800	Jia 2017
miR-1915-3p	up						
miR-187-3p	up						
miR-4485	up						
miR-4284	up						
miR-4508	up						
miR-1973	up						
miR-663a	up						
miR-877-5p	up						
miR-3195	up						
miR-596	up						
miR-4516	up						
miR-4488	up						
miR-636	up						
miR-27b-3p*	down						
miR-150-5p	down						
miR-200b-3p	down						
miR-205-5p	down						
miR-342-3p	down						
miR-203	down						
miR-21	up	55	20 (NLN)	FF and FFPE	qRT-PCR	1	Liu 2017
miR-21	up	26	10 (NLN)	FFPE	qRT-PCR	1	Song 2017
miR-10a	down	9	9 (RLH)	FF	qRT-PCR	1	Fan 2016
miR-23a	up	104	28	FFPE	qRT-PCR	1	Wang 2014
mir-146b	down	106	30 (RLH)	FFPE	qRT-PCR	939	Wu 2014
mir-320d	down						
miR-4284	up	24	14 (NLN)	FFPE	array	3100 probes	Tamaddon 2016
miR-21-5p	up						
miR-142-3p	up						
miR-155-5p	up						
miR-3182	up						
miR-16-5p	up						
miR-142-5p	up						
miR-451a	up						
miR-29b-3p	up						
miR-4301	up						
miR-146a-5p	up						
miR-146b-5p	up						
miR-29a-3p	up						
miR-342-3p	up						
miR-17-5p	up						
miR-20a-5p	up						
miR-let7g-5p	up						
miR-23b-3p	up						
miR-19b-3p	up						
miR-15a-5p	up						
miR-491-3p	up						
miR-4484	down						
miR-143	down						
miR-125B-5p	down						
miR-30C-1-3p	down						
miR-4534	down						

miR-7491	down						
miR-711	down						
miR-5571	down						
miR-630-5p	down						
miR-483-5p	down						
miR-4741	down						
miR-4778-5p	down						
miR-3158-5p	down						
miR-320a	down						
miR-101	down						
miR-224	down	258	40 (NLN)	FFPE	qRT-PCR	1	Ni 2015
miR-155	up	22	6 (NLN)	Biopsie	qRT-PCR	1	Huskova 2015
miR-16-1	up	63	5 GCB cells	FF	qRT-PCR	11	Troppan 2015
miR-16-2	up						
miR-27a	up						
miR-103	up						
miR-185	up						
miR-199	up						
miR-497	up						
miR-21	up	200	11 (NT)	FFPE	qRT-PCR	3	Go 2015
miR-17-92**	up						
miR-155	up						
miR-126	up	92	15	FF	sequencing	miRNAome	Lim 2015
miR-10b	up						
miR-145	up						
miR-126	up						
miR-424	up						
miR-134	up						
miR-199a-2	up						
miR-127	up						
miR-379	up						
miR-127	up						
miR-199b	up						
miR-143	up						
miR-34a	up						
miR-143	up						
miR-144	up						
miR-199b	up						
miR-139	up						
miR-199a-2	up						
miR-130a	up						
miR-542	up						
miR-125a	up						
miR-218-2	up						
miR-99b	up						
miR-125b-2	up						
miR-10a	up						
miR-145	up						
miR-455	up						
miR-214	up						
miR-628	up						
miR-146b	up						
miR-100	up						
miR-497	up						
miR-1301	up						
miR-451a	up						
miR-1247	up						
miR-574	up						
miR-195	up						
miR-340	up						
miR-326	up						
miR-196a-2	up						
miR-146b	up						
miR-338	up						
miR-675	up						
miR-337	up						
miR-511-2	up						

miR-10393-3p	up						
miR-99a	up						
miR-22	up						
miR-let-7c	up						
miR-217	up						
miR-654	up						
miR-452	up						
miR-503	up						
miR-455	up						
miR-let-7b	up						
miR-let-7e	up						
miR-450a-2	up						
miR-136	up						
miR-let-7a-2	up						
miR-196b	up						
miR-22	up						
miR-362	up						
miR-224	up						
miR-203a	down						
miR-205	down						
miR-20b	down						
NOVELM00290	down						
miR-4491	down						
miR-3150b	down						
miR-28	down						
miR-20b	down						
miR-28	down						
miR-129-2	down						
miR-3917	down						
miR-10392-5p	down						
miR-363	down						
miR-486	down						
miR-3934	down						
miR-138-1	down						
miR-3681	down						
NOVELM00288	down						
miR-138-1	down						
miR-27a	down						
miR-23a	down						
miR-589	down						
miR-10397-5p	down						
miR-4746	down						
miR-151a	down						
NOVELM00113	down						
miR-331	down						
miR-17	down						
miR-942	down						
miR-629	down						
miR-128-2	down						
miR-15b	down						
miR-3615	down						
miR-200b	down						
miR-181a-1	down						
miR-130b	down						
miR-582	down						
miR-616	down						
miR-185	down						
miR-200c	NS	61	13 (NLN)	Tissue	qRT-PCR	1	Berglund 2013
miR-150	down	45 (DC); 75 (VC)	10 (DC); 6 (VC)(NLN)	FF and FFPE	qRT-PCR/array	177	Caramuta 2013
miR-29b	down						
miR-29a	down						
miR-142-3p	down						
miR-142-5p	down						
miR-145	down						
miR-143	down						
miR-195	down						
miR-497	down						

miR-494	up						
miR-638	up						
miR-21	up	12	7	FFPE	qRT-PCR	4	Handal 2013
miR-155	up						
miR-16	down						
miR-150	up						
miR-155	up	90	31 (RLN)	FFPE	qRT-PCR	2	Zhong 2012
miR-146a	up						
miR-18b	up	36	5 (NLN)	Tissue	qRT-PCR	8	Fassina 2012
miR-19b	up						
miR-20a	up						
miR-92	up						
miR-93	up						
miR-106a	up						
miR-150	down						
miR-210	up						
miR-210	up	58	7 (NLN)	FFPE	qRT-PCR	157	Roehle 2008
miR-155	up						
miR-106a	up						
miR-17-5p	up						
miR-150	down						
miR-145	down						
miR-328	down						
miR-139	down						
miR-99a	down						
miR-10a	down						
miR-95	down						
miR-149	down						
miR-let7e	down						
miR-320	down						
miR-151	down						
miR-21	up	48	6 (NBC)	FF and FFPE	qRT-PCR	3	Lawrie 2007
miR-155	up						
miR-221	up						
miR-155	up	23	2	FF	Semi RT-PCR	1	Eis 2005

*expresion of miR-27b-3p was confirmed by qRT-PCR assays in 202 DLBCL and 10 RLH samples. **miR-17-92 cluster. RLH: Reactive lymphoid hyperplasia; NLN: normal lymph node tissues; NT: normal tonsil; FF: fresh frozen; FFPE: formalin-fixed paraffin-embedded; DC:discovery cohort; VC: validation cohort; NBC: normal B cell samples.

Table S2. Differentially expressed microRNAs in DLBCL subtypes.

Significant miRNAs	Result	n GBC	n ABC	Sample source	Method	Nº miRNAs	Reference
miR-21	Down GCB	19	36	FF and FFPE	qRT-PCR	1	Liu 2017
0	-	29	29	FF	qRT-PCR	1	Marques 2016
0	-	6	15	Biopsie	qRT-PCR	1	Huskova 2015
miR-155	Down GCB	53	95	FFPE	qRT-PCR	8	Go 2015
miR-28-3p	Up GCB	32	27	FFPE	qRT-PCR/array	377	Iqbal 2015
miR-28-5p	Up GCB						
miR-331-5p	Up GCB						
miR-589	Up GCB						
miR-129-3p	Up GCB						
miR-597	Up GCB						
miR-542-3p	Down GCB						
miR-155	Down GCB						

mir-1270	Up GCB						
mir-129-1-3p	Up GCB						
mir-129-2-3p	Up GCB						
mir-129-5p	Up GCB						
mir-138-1-3p	Up GCB						
mir-138-5p	Up GCB						
mir-151a-3p	Up GCB						
mir-151b	Up GCB						
mir-181a-5p	Up GCB						
mir-196b-5p	Up GCB						
mir-210-3p	Up GCB						
mir-28-3p	Up GCB						
mir-28-5p	Up GCB						
mir-301a-5p	Up GCB						
mir-3074-5p	Up GCB						
mir-30e-3p	Up GCB						
mir-3150b-3p	Up GCB						
mir-331-3p	Up GCB						
mir-339-3p	Up GCB						
mir-3681-5p	Up GCB						
mir-3934-3p	Up GCB	41	30	FF	sequencing	miRNAome	Lim 2015
mir-423-3p	Up GCB						
mir-4746-5p	Up GCB						
mir-582-3p	Up GCB						
mir-582-5p	Up GCB						
mir-589-3p	Up GCB						
mir-589-5p	Up GCB						
mir-598-3p	Up GCB						
mir-664a-3p	Up GCB						
mir-744-5p	Up GCB						
mir-10397-5p	Down GCB						
mir-106a-5p	Down GCB						
mir-10b-5p	Down GCB						
mir-148a-5p	Down GCB						
mir-155-3p	Down GCB						
mir-155-5p	Down GCB						
mir-17-5p	Down GCB						
mir-20a-5p	Down GCB						
mir-21-3p	Down GCB						
mir-221-3p	Down GCB						
mir-222-3p	Down GCB						

mir-222-5p	Down GCB						
mir-29b-1-5p	Down GCB						
mir-30b-3p	Down GCB						
mir-30d-3p	Down GCB						
mir-320a	Down GCB						
mir-363-3p	Down GCB						
mir-424-5p	Down GCB						
mir-503-5p	Down GCB						
mir-625-3p	Down GCB						
mir-625-5p	Down GCB						
mir-92a-1-5p	Down GCB						
NOVELM00288	Down GCB						
0	-	140	118	FFPE	qRT-PCR	1	Ni 2015
miR-199a<br>miR-497	Up GCB<br>Up GCB	36	17	FF	qRT-PCR	11	Troppan 2015
miR-320d	Down GCB	47	59	FFPE	qRT-PCR	2	Wu 2014
miR-155<br>miR-146a	Down GCB<br>Down GCB	20	34	FF and FFPE	qRT-PCR/array	177	Caramuta 2013
miR-155	Down GCB	36	31	FF	qRT-PCR	1	Huang 2012
0	-	10	8	FF	qRT-PCR	2	Kim 2012
miR-155<br>miR-146a	Down GCB<br>Down GCB	21	69	FFPE	qRT-PCR	2	Zhong 2012
miR-331<br>miR-151<br>miR-28<br>miR-454-3p<br>miR-222<br>miR-144<br>miR-451<br>miR-221	Up GCB<br>Up GCB<br>Up GCB<br>Up GCB<br>Down GCB<br>Down GCB<br>Down GCB<br>Down GCB	11	18	FFPE	qRT-PCR/array	470	Montes-Moreno 2011
0	-	8	17	FFPE	qRT-PCR	3	Nie 2010
miR-129<br>miR-138<br>miR-199b<br>miR-421<br>miR-520h<br>miR-569<br>miR-616<br>miR-620<br>miR-653	Up GCB<br>Up GCB<br>Up GCB<br>Up GCB<br>Up GCB<br>Up GCB<br>Up GCB<br>Up GCB<br>Up GCB	32	28	FFPE	Array	464	Lawrie 2009

miR-132	Down GCB						
miR-146b	Down GCB						
miR-155	Down GCB						
miR-186	Down GCB						
miR-190	Down GCB						
miR-194	Down GCB						
miR-21	Down GCB						
miR-213	Down GCB						
miR-221	Down GCB						
miR-222	Down GCB						
miR-301	Down GCB						
miR-30d	Down GCB						
miR-340	Down GCB						
miR-363	Down GCB						
miR-422b	Down GCB						
miR-518a	Down GCB						
miR-660	Down GCB						
miR-106b	NA						
miR-140-3p	NA						
miR-142-3p	NA						
miR-142-5p	NA						
miR-151-5p	NA						
miR-16	NA						
miR-184	NA						
miR-191	NA						
miR-19a	NA						
miR-19b	NA	20	20	Tissue	Array	113	Zhang 2009
miR-20a	NA						
miR-28-5p	NA						
miR-299-5p	NA						
miR-30c	NA						
miR-30e	NA						
miR-30e*	NA						
miR-32	NA						
miR-526b*	NA						
miR-583	NA						
miR-129	NA						
miR-133a	NA						
miR-133b	NA	25	25	FFPE	qRT-PCR	157	Roehle 2008
miR-138	NA						
miR-151	NA						

miR-155	NA						
miR-199b	NA						
miR-27b	NA						
miR-155	Down GCB						
miR-21	Down GCB	16	18	FF and FFPE	qRT-PCR	3	Lawrie 2007
miR-221	Down GCB						
miR-155	Down GCB	4	19	FF	Semiq. RT-PCR	1	Eis 2005

GCB: Germinal center B-cell like; FF: fresh frozen; FFPE: formalin-fixed paraffin-embedded; NA: not available.

Table S3. Differentially expressed microRNAs as prediction to response to R-CHOP therapy.

Significant miRNAs	Result	n DLBCL	Sample source	Method	Nº miRNAs	Reference
miR-27	FR	201	Tissue	nanostring	800	Jia 2017
miR-34a	FR	62	FF	qRT-PCR	1	Marques 2016
0	-	22	Biopsie	qRT-PCR	1	Huskova 2015
miR-224	FR	258	FFPE	qRT-PCR	1	Ni 2015
hsa-miR-155<br>hsa-miR-146	UFR<br>UFR	90	FFPE	qRT-PCR	2	Zhong 2012

UFR: unfavorable response; FR: favorable response; FF: fresh frozen; FFPE: formalin-fixed paraffin-embedded.

Table S4. Differentially expressed microRNAs as prognosis biomarkers.

Significant miRNAs	Result	n DLBCL	Sample source	Method	Nº miRNAs	Reference
miR-27b	Down: ↓ OS	202	Tissue	qRT-PCR	1	Jia 2017
miR-34a	Up: ↑ OS	62	FF	qRT-PCR	1	Marques 2016
miR-155	Up: lower survival	118	FF	qRT-PCR	1	Zhu 2016
miR-23a	Up: ↓ OS	104	FFPE	qRT-PCR	1	Wang 2014
miR-155<br>miR-16<br>miR-363<br>miR-24	Up: ↓ OS<br>Up: ↓ OS<br>Up: ↓ OS<br>Up: ↑ OS	79	FFPE	qRT-PCR	8	Iqbal 2015
miR-214-5p<br>miR-28-5p<br>miR-324-5p<br>miR-339-3p<br>miR-5586-5p<br>NOVELM00203M	Up: ↑ OS and EFS<br>Up: ↑ OS and EFS<br>Up: ↓ OS and PFS<br>Up: ↑ OS and EFS<br>Up: ↑ OS and EFS<br>Up: ↓ OS and PFS	92	FF	sequencing	miRNAome	Lim 2015
miR-224	Up: ↑ OS and PFS	258	FFPE	qRT-PCR	1	Ni 2015
miR-17-5p<br>miR-19-3p<br>miR-20a-5p<br>miR-106a-5p<br>miR-150-5p*<br>miR-342-3p*<br>miR-181a-5p<br>miR-140-3p	Up: poor prognosis<br>Up: poor prognosis<br>Up: poor prognosis<br>Up: poor prognosis<br>Down: poor prognosis<br>Down: poor prognosis<br>Down: poor prognosis<br>Down: poor prognosis	83	FFPE	qRT-PCR/array	±900	Shepshelovich 2015
miR-199a	Up: ↑ OS and DFS	58	FF	qRT-PCR	11	Troppan 2015

miR-497	Up: ↑ OS and DFS					
miR-17-92	Up: ↓ OS and PFS	200	FFPE	qRT-PCR	3	Go 2015
miR-21	Up: ↓ OS and PFS					
miR-146b-5p	Down: ↓ PFS	12 (DC); 106 (VC)	FFPE	qRT-PCR	939	Wu 2014
miR-320d	Down: ↓ PFS and OS					
miR-200c	Up: ↓ OS	61	Tissue	qRT-PCR	1	Berglund 2013
miR-146a	Down: ↑ PFS	90	FFPE	qRT-PCR	2	Zhong 2012
miR-155	Down: ↑ PFS					
miR-181	Up: ↑ PFS	176	FFPE	qRT-PCR	11	Alencar 2011
miR-18a	Up: ↓ OS					
miR-222	Up: ↓ OS					
miR-221	Up: ↑ PFS and OS	36/240	FFPE	qRT-PCR/array	470/9	Montes-Moreno 2011
miR-331	Up: ↑ PFS and OS					
miR-222	Up: ↓ PFS and OS					
miR-93	Up: ↓ PFS and OS					
miR-148a	NA					
miR-151	NA					
miR-28	NA					
miR-451	NA					
miR-491	NA					
miR-100	Up: ↑ EFS	64	FFPE	Array	464	Lawrie 2009
miR-199a*	Up: ↑ EFS					
miR-199b	Up: ↑ EFS					
miR-23a	Up: ↑ EFS					
miR-24	Up: ↑ EFS					
miR-27a	Up: ↑ EFS					
miR-30e	Up: ↑ EFS					
miR-330	Up: ↑ EFS					
miR-425	Up: ↑ EFS					
miR-302	Up: ↓ EFS					
miR-608	Up: ↓ EFS					
miR-637	Up: ↓ EFS					
miR-222	Up: ↓ OS and PFS	106	FFPE	qRT-PCR	3	Malumbres 2009
let-7g	Down: ↑ EFS	58	Biopsie	qRT-PCR	157	Roehle 2008
miR-195	Down: ↑ EFS					
miR-19a	Down: ↑ OS					
miR-21	Down: ↑ OS					
miR-23a	Down: ↑ OS					
miR-27a	Down: ↑ OS					
miR-34a	Down: ↑ OS					
miR-127	Up: ↓ OS and PFS					
miR-21	Up: ↑ RFS	35	FF and FFPE	qRT-PCR	3	Lawrie 2007

*were validated in an independent set of 13 samples FF: fresh frozen; FFPE: formalin-fixed paraffin-embedded;OS:overall survival, PFS: progression-free survival; EFS: event free survival;RFS:relapse free survival.

Table S5. Pathways significantly enriched among hsa-miR-155-5p and hsa-miR-21-5p (DLBCL diagnosis) target genes predicted by 6 or more databases.

miRNA	pathway name	set size	candidates contained	p-value	FDR	pathway source
**miR-155-5p miR-21-5p (2644 target genes)**	Diseases of signal transduction	247	68 (27.5%)	1.03e-08	1.76e-05	Reactome
	**MAPK signaling pathway-Homo sapiens (human)**	**295**	**73 (24.7%)**	**3.42e-07**	**0.000293**	**KEGG**
	Signaling by Receptor Tyrosine Kinases	422	94 (22.3%)	1.34e-06	0.000767	Reactome

	Pathways in cancer - Homo sapiens (human)	526	111 (21.1%)	2.45e-06	0.00105	KEGG
	Prolactin signaling pathway - Homo sapiens (human)	70	24 (34.3%)	1.25e-05	0.00295	KEGG
	IRS-related events trigged by IGF1R	49	19 (38.8%)	1.35e-05	0.00295	Reactome

set size: # of genes in the pathway; candidates contained:# of gene targets in the pathway; p-value: absolute p-value; FDR: corrected p-value by "False Discovery Rate" method; pathway source: associated database.

Table S6. Pathways significantly enriched among hsa-miR-155-5p, hsa-miR-221-3p (DLBCL subtype) target genes predicted by 6 or more databases.

miRNA	pathway name	set size	candidates contained	p-value	FDR	pathway source
**miR-155-5p miR-221-3p (2793 target genes)**	Membrane Trafficking	582	145 (24.9%)	5.34e-11	9.16e-08	Reactome
	Vesicle-mediated transport	422	103 (24.4%)	1.09e-07	6.25e-05	Reactome
	**Signaling by Receptor Tyrosine Kinases**	422	103 (24.4%)	1.09e-07	6.25e-05	Reactome
	Diseases of signal transduction	247	67 (27.1%)	3.51e-07	0.000136	Reactome
	Signaling pathways regulating pluripotency of stem cells - Homo sapiens (human)	139	44 (31.7%)	3.98e-07	0.000136	KEGG
	Gene expression (Transcription)	1372	266 (19.4%)	8.21e-07	0.000189	Reactome

References

1

2

© 2019 by the authors. Submitted for possible open access publication under the terms and conditions of the Creative Commons Attribution (CC BY) license (http://creativecommons.org/licenses/by/4.0/).
